# Physiological vortices in the sinuses of Valsalva: An *in vitro* approach for bio-prosthetic valves

**DOI:** 10.1016/j.jbiomech.2016.05.027

**Published:** 2016-09-06

**Authors:** Riccardo Toninato, Jacob Salmon, Francesca Maria Susin, Andrea Ducci, Gaetano Burriesci

**Affiliations:** a UCL Cardiovascular Engineering Laboratory, UCL Mechanical Engineering, University College London, UK; b Cardiovascular Fluid Dynamics Laboratory HER, Department of Civil, Environmental and Architectural Engineering – University of Padua, Italy

**Keywords:** Valsalva sinuses, Bioprosthetic valve, PIV, *In vitro* testing

## Abstract

**Purpose:**

The physiological flow dynamics within the Valsalva sinuses, in terms of global and local parameters, are still not fully understood. This study attempts to identify the physiological conditions as closely as possible, and to give an explanation of the different and sometime contradictory results in literature.

**Methods:**

An *in vitro* approach was implemented for testing porcine bio-prosthetic valves operating within different aortic root configurations. All tests were performed on a pulse duplicator, under physiological pressure and flow conditions. The fluid dynamics established in the various cases were analysed by means of 2D Particle Image Velocimetry, and related with the achieved hydrodynamic performance.

**Results:**

Each configuration is associated with substantially different flow dynamics, which significantly affects the valve performance. The configuration most closely replicating healthy native anatomy was characterised by the best hemodynamic performance, and any mismatch in size and position between the valve and the root produced substantial modification of the fluid dynamics downstream of the valve, hindering the hydrodynamic performance of the system. The worst conditions were observed for a configuration characterised by the total absence of the Valsalva sinuses.

**Conclusion:**

This study provides an explanation for the different vortical structures described in the literature downstream of bioprosthetic valves, enlightening the experimental complications in valve testing. Most importantly, the results clearly identify the fluid mechanisms promoted by the Valsalva sinuses to enhance the ejection and closing phases, and this study exposes the importance of an optimal integration of the valve and root, to operate as a single system.

## 1 Introduction

The formation of vortical structures into the Valsalva sinuses was already reported in the early XVI century (Keele, 1952, Robicsek, 1991). After the 60s, the development of heart valve substitutes stimulated new research in the cardiovascular community towards a better understanding of the flow dynamics occurring in the aortic root (Bellhouse and Bellhouse, 1968, Bellhouse and Talbot, 1969, van Steenhoven and van Dongen, 1979). It is now well accepted that the presence of the Valsalva sinuses influences the dynamics of the valve leaflets, and plays a relevant role in the washout of the sinus flow structures and in the blood supply of the coronaries (van Steenhoven et al., 1982; Peskin, 1982; Peacock, 1990; Rubenstein et al., 2012; Caro et al., 2012). However, the various and often contradictory interpretations provided in the literature for the fluid dynamics established within the sinuses (Leo et al., 2006, Yap et al., 2012, Moore and Dasi, 2014) still reveal a lack of understanding of the physiological flow conditions that these chambers promote in the aortic root.

**Fig. 1:**
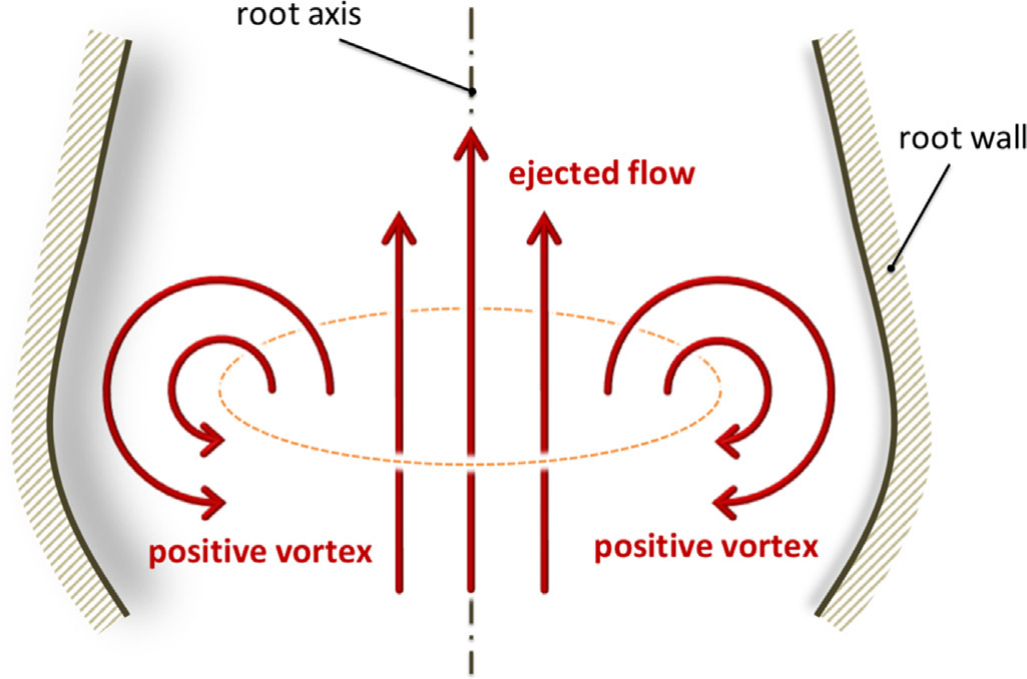
Sketch describing a positive vortex ring formed during the ejection phase.

A first mechanism was proposed by Bellhouse and Bellhouse (1968), who suggested that the sinuses have the function to host and expand the start-up vortex ring that generates at the valve exit during early systole, with the vortex also promoting leaflet closure. This vortex follows the ejected flow in proximity of the root axis, and has opposite direction close to the arterial wall (to avoid any ambiguity, this vortical rotation, as sketched in Fig. 1, will be denoted as positive in the remainder of the paper). The vortex dynamics identified by Bellhouse have been confirmed by a number of numerical studies from different groups (Swanson and Clark, 1973, de Hart, 1997, de Hart et al., 2003, Korakianitis and Shi, 2006, Katayama et al., 2008), and are observed in recent *in vivo* works based on high intensity Magnetic Resonance Imaging (MRI) (Yang et al., 1998, Escobar Kvitting et al., 2004, Markl et al., 2005, Ranga et al., 2006, Markl et al., 2011), though the poor spatial and temporal resolutions of this measurement technique are insufficient to provide a conclusive answer on the dynamics.

Despite the general consensus on the presence of a vortex ring in the valve opening stage, recent *in vitro* investigations have reported more complex fluid dynamics, where the start-up vortex ring generated during valve opening is convected away towards the aorta, and a secondary vortex with opposite rotation forms and remains within the sinus until the valve begins to close (Leo et al., 2005, Leo et al., 2006, Dasi et al., 2009, Saikrishnan et al., 2012, Ducci et al., 2013). The numerical simulations of Fukui and Orinishi (2013) also reported the presence of multiple vortices within each sinus during the cardiac cycle, which depended on variation of the Valsalva Sinuses morphology (extension and depth).

This study provides an in depth investigation of the hemodynamics occurring within the aortic root, proposing justifications for the different flow modalities reported in previous studies. In particular, various combinations of aortic root geometries and prosthetic valves are studied *in vitro* on a pulse duplicator, using 2D Particle Image Velocimetry (PIV) to analyse the local flow characteristics. The different configurations are selected to reproduce idealised physiological conditions, as well as the common departures from clinical configurations often introduced in the described studies, such as mismatch of valve-root size, variations in the axial position of the valve (*e.g*. infra or supra-annular implant) and absence of the sinuses (as in valved-grafts).

## 2 Material and methods

In this study, all experiments were carried out using a hydro-mechanical pulse duplicator (Vivitro Superpump System SP3891, ViVitro Labs Inc., Canada), with pressure catheters monitoring the pressure upstream and downstream of the aortic valve. These, combined with the instantaneous volumetric flowrate, allowed estimation of the following characteristic parameters: the pressure drop across the valve, the effective orifice area of the valve, the energy loss of the valve, and the closing volume. Tests were performed imposing a physiological flowrate of 4 l/min, a heart rate of 70 bpm with 35% of systolic time, and a mean aortic pressure of 100 mmHg. The mean and standard deviation (±SD) of the estimated parameters are reported in Table 1. Further information about the testing instrumentation is provided in the Appendix.

**Fig. 2:**
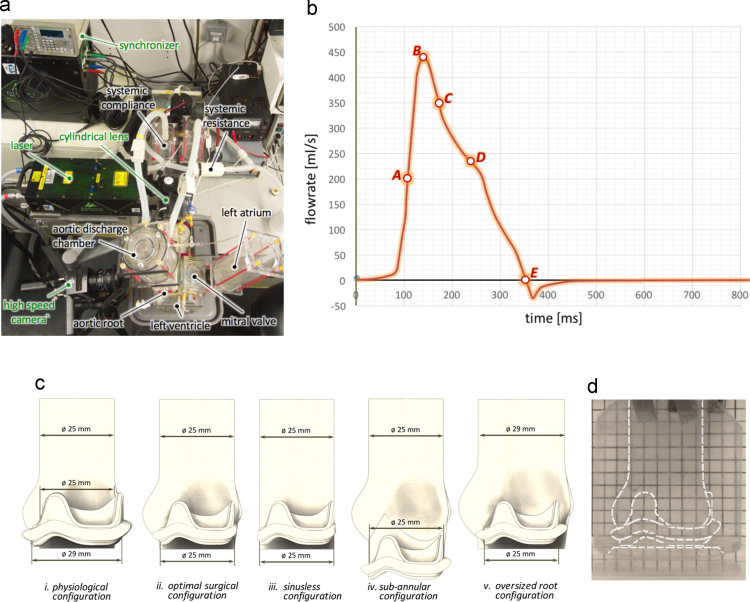
Top view of the system setup (a); typical diagram of the flowrate vs. time during a heart cycle with analysed instants (b); valve-root configurations considered in the study (c); and grid system used to verify refractive index matching of the silicone root and the work solution (here reported for the physiological configuration) (d).

Local fluid dynamics were investigated by means of 2D PIV, which is a laser based, non-intrusive optical technique, providing measurements of instantaneous velocity vector fields by correlating the displacement of seeding particles on a laser plane over a time interval ∆*t*, selected to catch the flow features of each of the analysed instants. The system set up is represented in Fig. 2a, where the positions of the camera and laser with respect to the valve root configuration are represented. Measurements were carried out on a root cross section (sagittal plane), bisecting one of the sinuses. A phase-resolved approach was selected to analyse the PIV data, because it allowed to meet the main objective of the study, which is to identify and compare the large scale flow features for different valve-root configurations. Camera and laser were synchronised with the pulse duplicator, and five reference instants associated with specific flow features were selected to characterise the hemodynamics of the valve-root configurations during each cardiac cycle. It should be noted that the reference instants can occur at different times of the cycle for the different valve-root setups. The reference instants, represented in Fig. 2b on the diagram of the cyclic flowrate obtained for the optimal surgical configuration (the features allowing the identification of the reference instants were similar for all studied configurations), correspond to the times when the ejected flow exhibits/reaches the following conditions:

A) maximum increasing flowrate;
B) peak flowrate;
C) maximum decreasing flowrate;
D) most significant change of curvature in the decreasing region;
E) zero flowrate.

Further information on the PIV settings used is provided in the Appendix.

A set of mock aortic roots was built to assess the impact of the sinuses and of the aortic root proportions on the flow downstream of the valve, and replicate common testing arrangements. A reference diameter of 25 mm at the sino-tubular junction (STJ) was selected, as this is representative of an average size for adult humans (Davis et al., 2014). An additional root was manufactured, where the STJ size was increased to 29 mm, and used to verify the effect of valve undersizing. Valsalva sinuses were modelled based on the geometric proportions described by Swanson and Clark (1973), the epitrochoidal top view profile defined by Reul et al. (1990), the leaflet angles identified by Thubrikar et al. (1981), and the sagittal plane sinus profile suggested by Grigioni et al. (2005). All roots were made of optically clear, solvent free, low viscosity silicone elastomer (MED-6015, NuSil Technology, CA, USA, refractive index *n*=1.4). For this study, it was preferred to opt for thick-wall roots, with negligible compliance. Though this is an approximation, the root elasticity depends on a number of factors including the geometry and materials of the chamber and, *in vivo*, the age and healthiness of the tissues or, in the presence of a graft, the prosthetic materials and its degree of cellular infiltration. Hence, it was decided to exclude this variable.

To reduce optical distortion, the refractive index of the working fluid was matched to that of the silicon root by adding potassium iodide (KI) to distilled water until the distortion of a grid placed at the back of the silicone root was minimal (see Fig. 2d). Due to the large number of comparative experiments requiring the same bioprosthetic valve, it was preferred not to match blood viscosity by adding glycerine, so to avoid any change in the tissue mechanical properties which could have made the comparison of the different sets of results ineffective (Wright, 1979). Though this approximation is accepted by international regulations for testing of bioprosthetic valves (ISO 5840, 2009), it may result in some departure from the physiological behaviour.

For the valve model, porcine bioprostheses were preferred, due to their similarity with healthy human aortic valves in terms of shape, thickness and material (Thubrikar, 1990). In particular, LabCorp TLPB stented porcine surgical prostheses of size 25 (*i.e*. 25 mm external stent diameter) and size 29 (*i.e*. about 25 mm internal diameter) were used. The size 25 valve was chosen to achieve optimum surgical matching of the device with the 25 mm roots, whilst the larger valve uses leaflets extracted from a porcine aortic root with a STJ diameter equal to about 25 mm, providing a better description of the native anatomy.

The following five different valve-root configurations were studied, as illustrated in Fig. 2c:

i) *physiological configuration*: 29 mm valve (25 mm leaflets) in aortic root (including sinuses) of STJ diameter equal to 25 mm with a groove to host the stent thickness – describes an idealised healthy native situation, where the influence of the stent is minimised.
ii) *optimal surgical configuration*: 25 mm valve in aortic root (including sinuses) of STJ diameter equal to 25 mm – describes an optimum implantation of the prosthetic valve in a supra-annular position;
iii) *sinusless surgical configuration*: 25 mm valve in straight cylindrical root of diameter equal to 25 mm – describes the flow in the absence of sinuses;
iv) *sub-annular configuration*: 25 mm valve in aortic root (including sinuses) of STJ diameter equal to 25 mm – describes an infra-annular implantation of the prosthetic valve (8.5 mm below the ideal position), and is the default positioning for the valve housing in many commercial pulse duplicators (Lim et al., 1997, Lim et al., 1998, Lim et al., 2001);
v) *oversized root configuration*: 25 mm valve in aortic root (including sinuses) of STJ diameter equal to 29 mm – describes an optimum implantation of the prosthetic valve in an enlarged root, common in testing situations (Leo et al., 2005, Leo et al., 2006, Dasi et al., 2009, Saikrishnan et al., 2012, Ducci et al., 2013).

## 3 Results

**Fig. 3:**
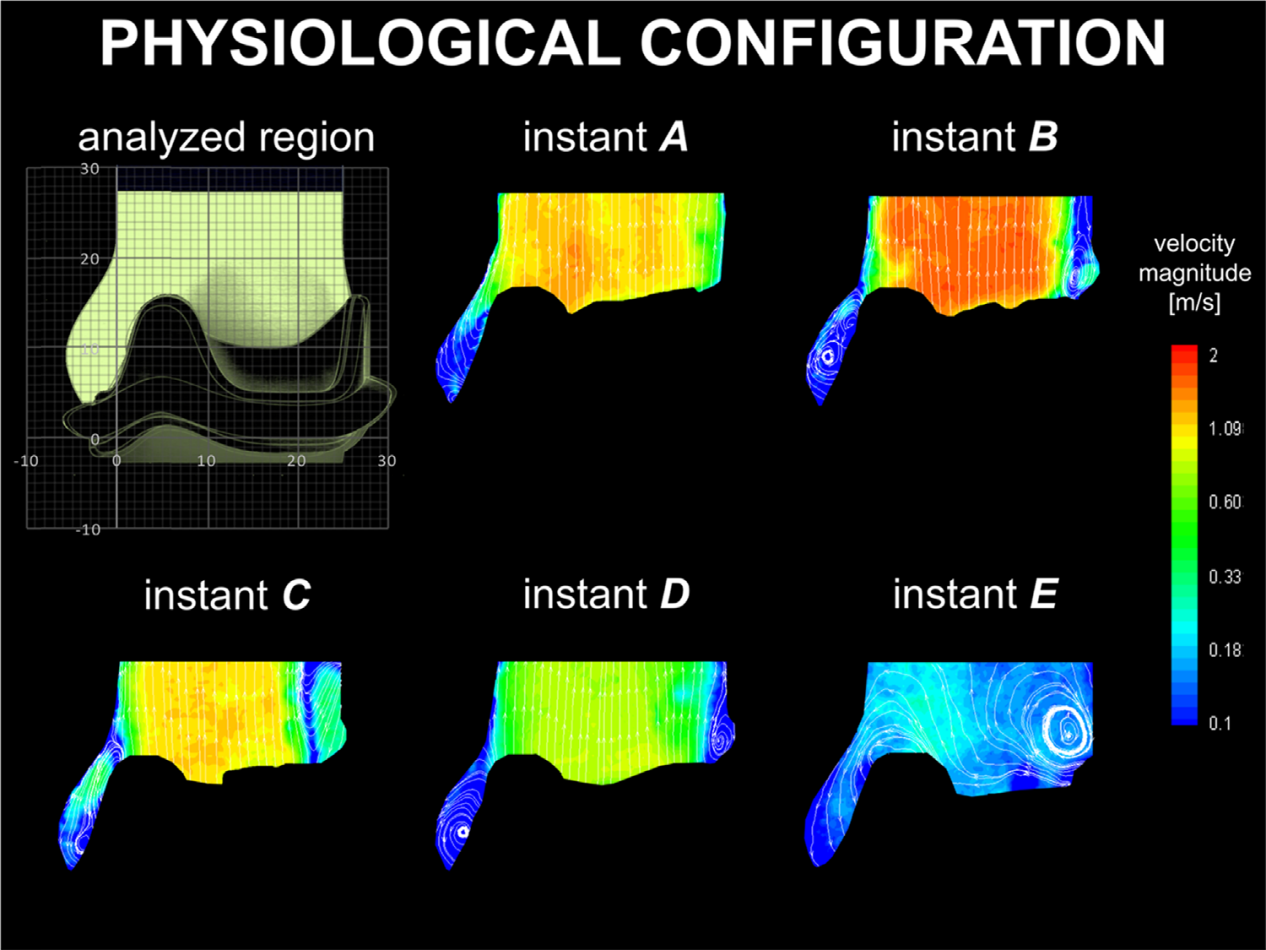
Velocity contour maps and streamlines for the physiological configuration.

**Fig. 4:**
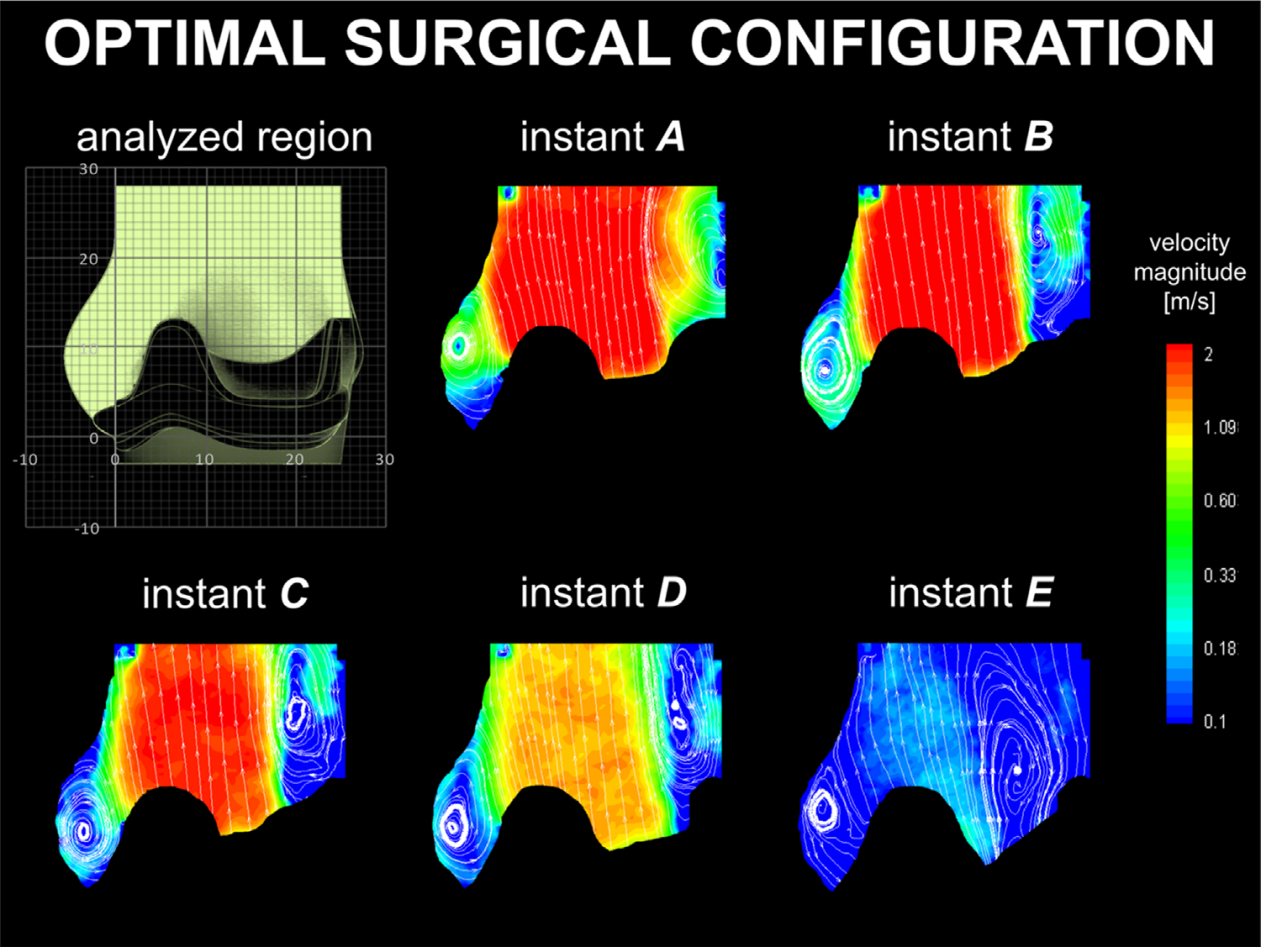
Velocity contour maps and streamlines for the optimal surgical configuration.

**Fig. 5:**
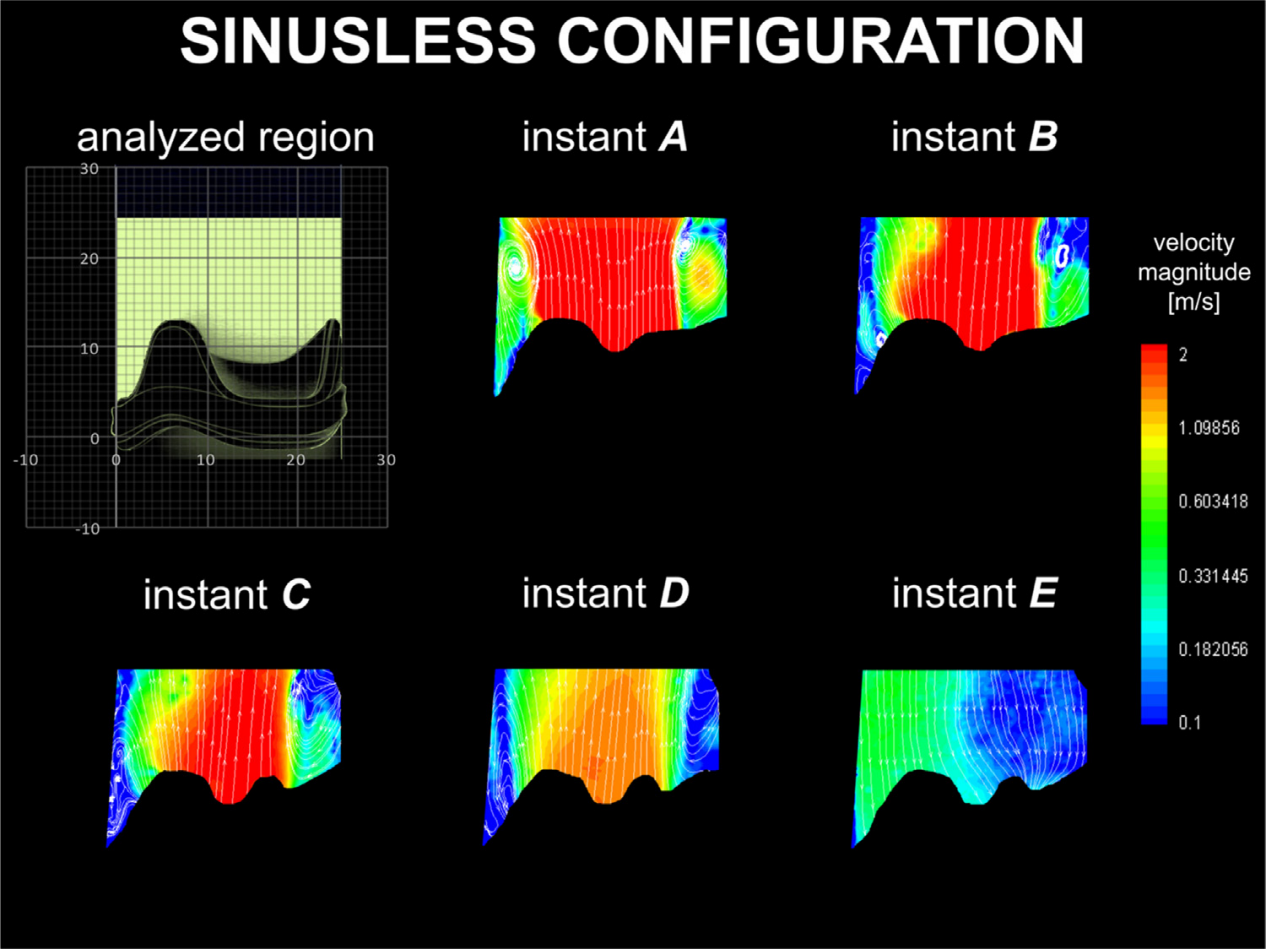
Velocity contour maps and streamlines for the sinusless surgical configuration.

**Fig. 6:**
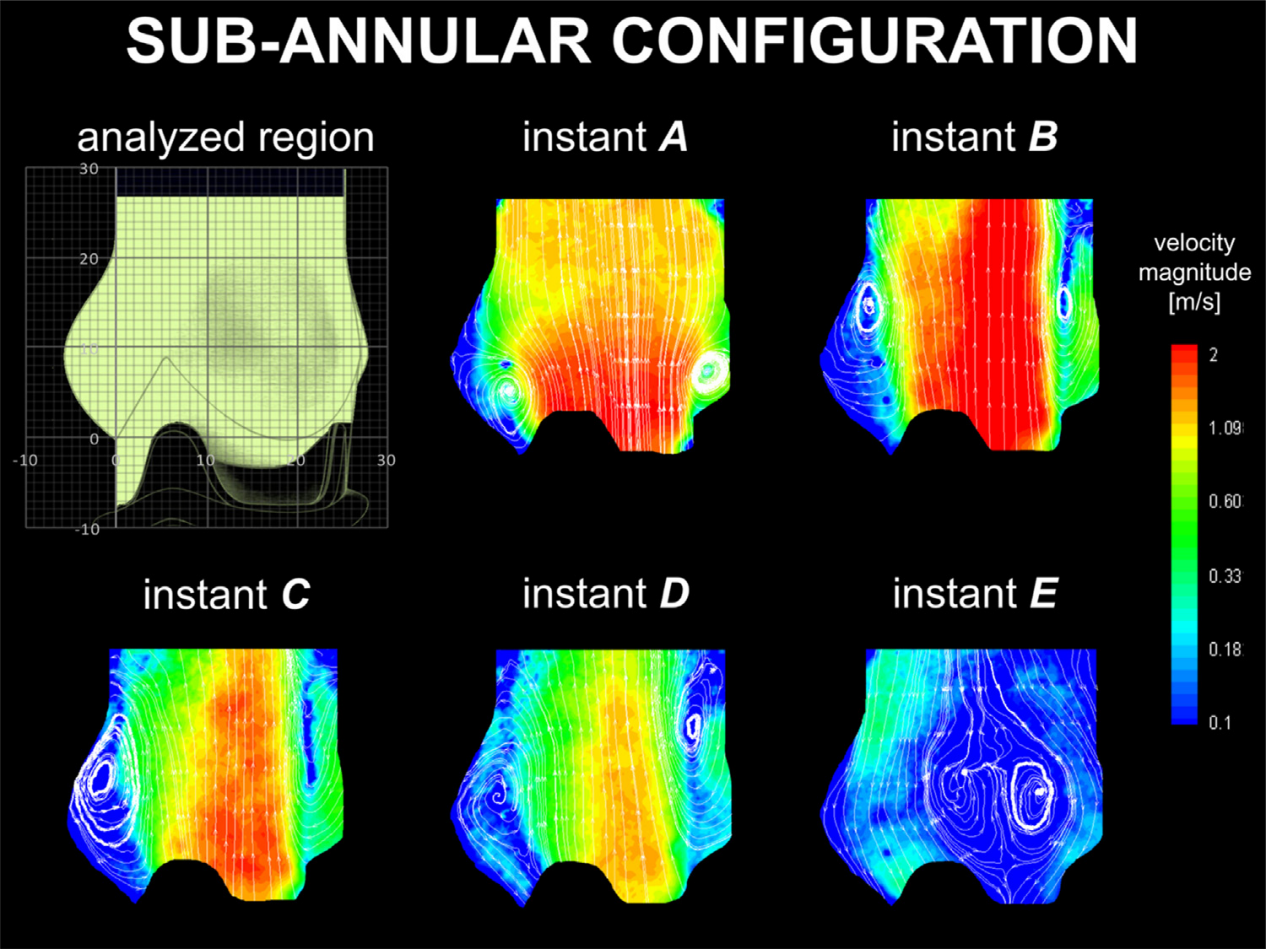
Velocity contour maps and streamlines for the sub-annular configuration.

**Fig. 7:**
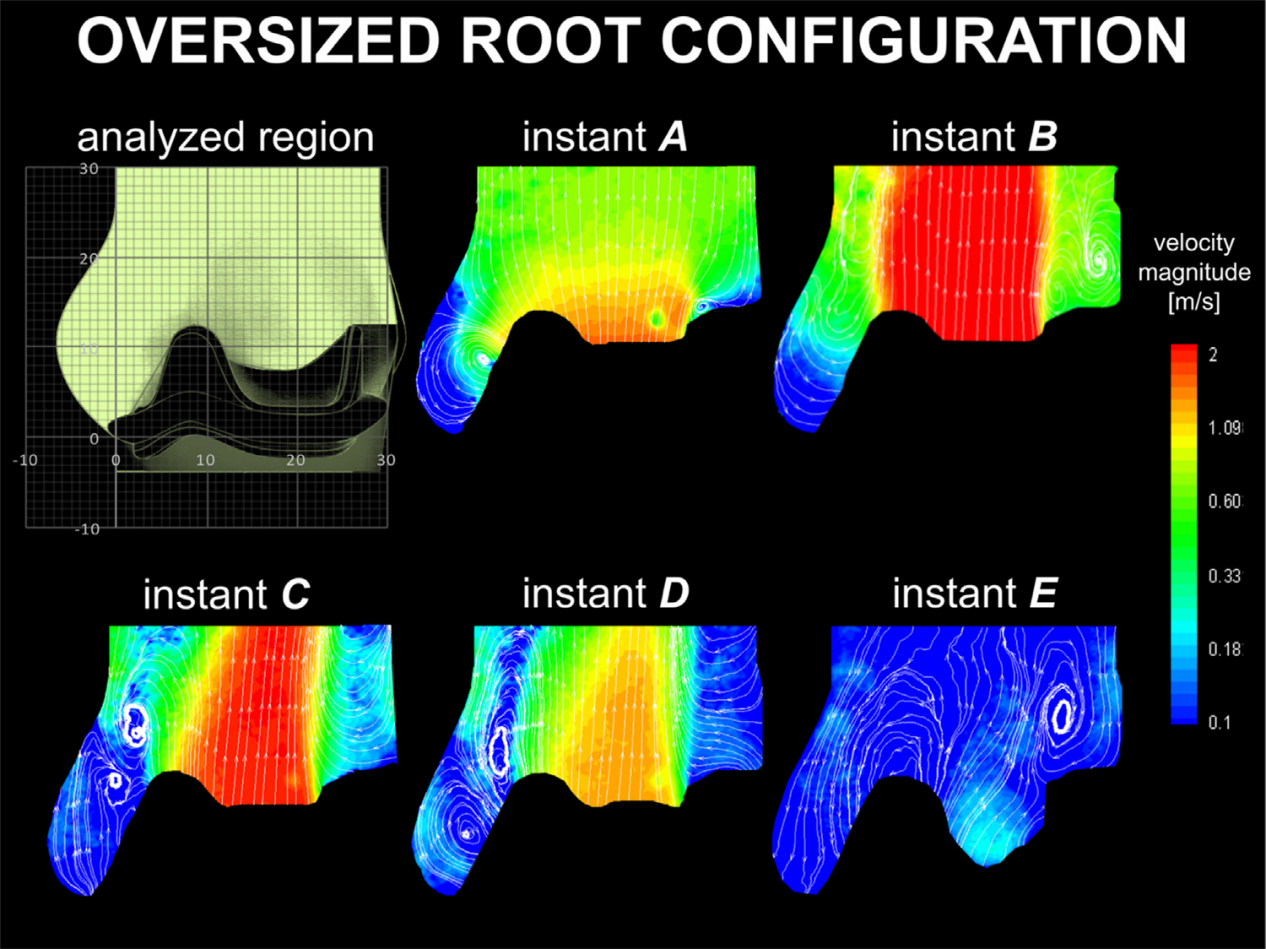
Velocity contour maps and streamlines for the oversized root configuration.

Below is provided a description of the main flow features for each valve-root configuration, with contour maps of the average velocity magnitude and corresponding streamlines for each case presented in Fig. 3, Fig. 4, Fig. 5, Fig. 6, Fig. 7. Maximum PIV velocity obtained in the measurement region (*V*_*max*_) and the diameter of the *vena contracta* (*d*_*vc*_), alongside the global hydrodynamic performances – quantified in terms of effective orifice area (*A*_*EO*_), mean systolic transvalvular pressure drop (Δ*p*), closing regurgitant volume (*R*_*c*_), and energy loss calculated as the sum of the forward and closing components (*E*_*loss*_) – are summarised in Table 1. The *vena contracta* is defined as the minimum width of the fast forward jet (*i.e*. higher than 1 m/s) at the maximum flow rate (instant *B*).

**Fig. 8:**
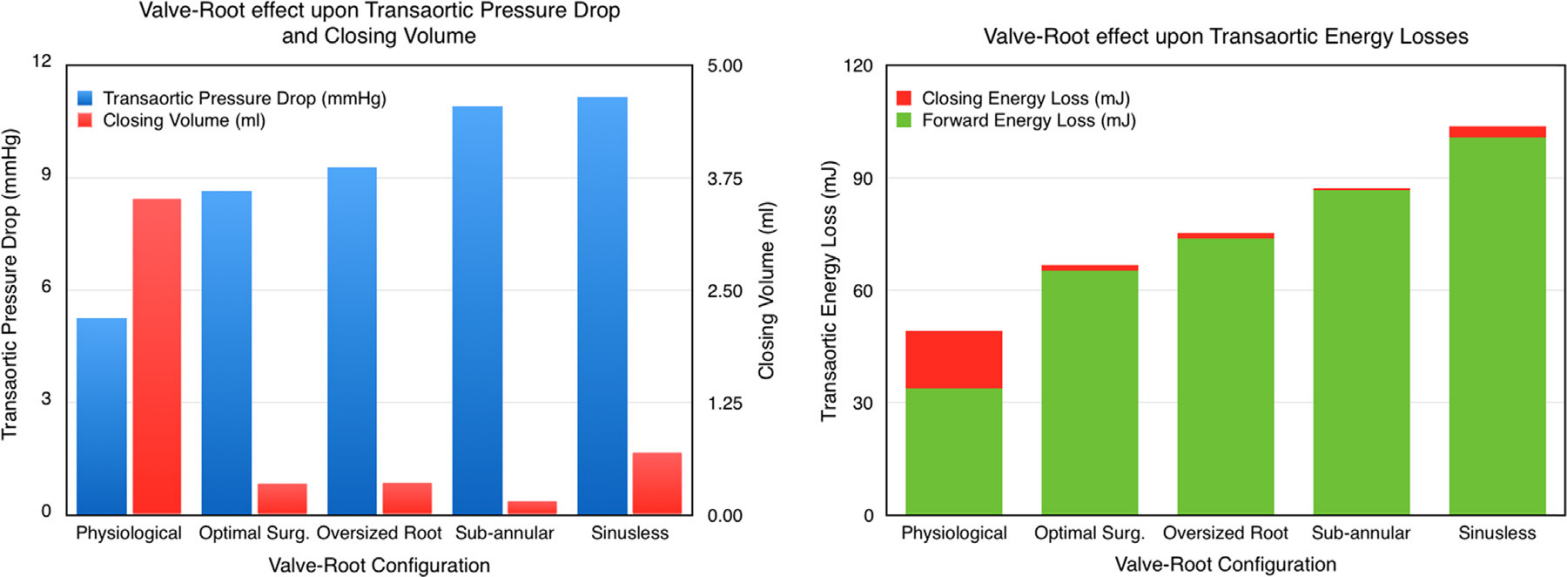
(a) Transaortic pressure drop and closing volume for each configuration; (b) Transaortic energy losses during forward flow and closing phases for each configuration.

Bar diagrams allowing a visual comparison of the variation of Δ*p* and *R*_*c*_, as well as the associated energy losses, for the various configurations are shown in Fig. 8.

For clarity, the flow behaviour of each case is compared with the physiological setup, as this reproduces the closest conditions to those expected for a healthy natural configuration.

### 3.1 Physiological configuration

The velocity maps for the physiological configuration are presented in Fig. 3. A positive start-up vortex forms at the beginning of the ejection phase, and is captured in the sinus throughout the systole, up to the early stages of diastole (instants *A–D* in Fig. 3), disappearing only after complete valve closure (instant *E* in Fig. 3). During opening the valve leaflets expand into the sinuses. The systolic jet takes a slightly divergent profile, which occupies most of the root section above the STJ, with the exception of a small vortical region forming above the leaflets׳ commissure (evident at instant *B* in Fig. 3).

### 3.2 Optimal surgical configuration

The velocity maps for the optimal surgical configuration are presented in Fig. 4. Similarly to the physiological case, a positive start-up vortex generates during valve opening and remains entrapped within the sinus throughout systole, losing its intensity during valve closure (instant *E* in Fig. 4). A second positive vortex develops above the leaflets׳ commissure at the beginning of systole, larger than that observed in the physiological configuration. This second vortex expands towards the centre of the root as the flow decreases (instant *E* in Fig. 4).

### 3.3 Sinusless configuration

The results from the sinusless configuration are described in Fig. 5. During systole, a positive start-up vortex ring generates at the exit of the opening leaflet, in close proximity to the aortic wall (instant *A* in Fig. 5, left side). Contrary to previous cases, this is carried away from the valve with the ejected flow (instants *A* and *B* in Fig. 5). This mechanism is most evident at the maximum forward flow (instant B), and repeats several times during the ejection phase, arresting only after valve closure (instant *E* in Fig. 5). As with the previous configuration, a positive vortical structure forms above the stent post and remains throughout systole, alongside a relatively narrow cross section of the main jet.

### 3.4 Sub-annular configuration

The velocity maps for the sub-annular configuration are represented in Fig. 6. At the beginning of systole, a positive start-up vortex ring develops at the base of the sinus and expands, moving towards the STJ at the flow peak (instant *A–B* in Fig. 6). During the decreasing flow phase, another positive vortex forms inside the sinus (instants *C–D* in Fig. 6), and dissipates during valve closure (instant *E* in Fig. 6). Similarly to the previous cases, a positive vortex of similar dimensions to the one observed in the optimal surgical case generates above the stent post (instants *B–D* in Fig. 6). This vortex expands towards the root axis during diastole (instant *E* in Fig. 6).

### 3.5 Oversized root configuration

The velocity maps obtained for the oversized root configuration are presented in Fig. 7. A positive start-up vortex forms inside the sinus during maximum forward flow, whilst the central jet spreads over the entire aortic cross section. This vortex soon escapes towards the ascending aorta and increases in intensity, stabilising in proximity to the STJ (instant *B–D* in Fig. 7). A secondary vortex ring with opposite direction (*i.e*. a negative vortex) generates inside the sinus, replacing the departed start-up vortex (instants *C*–*D* in Fig. 7). As with the optimal surgical and sub-annular cases, a positive vortex forms on top of the stent post (instants *B–E* in Fig. 7). This vortical structure moves up during systole, with its centre positioning about 2 cm above the distal portion of the stent, at the instant of maximum decreasing flowrate (instant *C* in Fig. 7).

## 4 Discussion

The physiological configuration, which best replicates healthy native anatomies, is characterised by a start-up vortex, which is entrapped within the sinus for most of systole. As a result, the ejected flow is relatively unrestricted as it spreads to the STJ, resulting in a more favourable ratio between *d*_*vc*_ and the valve inner diameter compared to all other cases.

In the optimal surgical configuration, a smaller valve of the same typology, providing ideal surgical matching inside the idealised root, is implanted in a supra-annular position. The opening mechanism is very similar, with the start up vortex captured and contained within the sinus soon after opening. However, due to the smaller leaflets and the presence of the stent inside the root lumen, the vortices in the sinus and at the commissures are larger, and constrain the systolic jet, decreasing the system performances. In particular, *A*_*EO*_ is 32% smaller than in the physiological configuration, corresponding to an increase of Δ*p* of about 64%, and resulting in a peak velocity 70% higher. The smaller valve is advantageous in the closing phase, which is associated with a 90% reduction in the closing regurgitant volume, due to the shorter distance the leaflets need to travel and the reduced geometric orifice area. However, this cannot compensate the systolic losses, and the total *E*_*loss*_ is 36% higher than for the physiological configuration.

Though the sinusless surgical configuration may appear to model an extreme scenario, it is similar to the clinical case of a valve implanted into a tubular aortic graft, and analogous to the case of transcatheter valves, for which the native leaflets and the supporting structure act as a containing tube (Ducci et al., 2013). This configuration is characterised by repeated formation and migration of vortices which constrict the main jet, producing an increase in *V*_*max*_ and Δ*p* of about 75% and 110%, respectively, and a reduction of *A*_*EO*_ of 36% compared to the physiological case. In fact, a major function of the sinuses appears to be to host the start-up vortex throughout systole, enabling the main jet to reattach to the wall at the STJ (Fig. 4). Moreover, the absence of the Valsalva chambers causes a significant decrease in the closing efficiency (as expressed by energy and closing parameters in Table 1) compared to the optimal surgical configuration, probably due to the reduction of the radial component of the flow, which is allowed to develop in the sinuses and promotes leaflets closure. Globally, the total energy loss is more than double (+111%) compared to the physiological configuration, and 55% higher than in the optimal surgical case. This corroborates the hypothesis that the root geometry and the presence of the Valsalva chambers have a major impact on the flow characteristics downstream of the valve (Bellhouse and Bellhouse, 1968, van Steenhoven et al., 1982, Peskin, 1982, Peacock, 1990). Improvements in the valve performances due to the presence of the Valsalva chambers have recently been reported in other *in vivo* and *in vitro* studies (Schoenhoff et al., 2009, Pisani et al., 2013), and the fluid dynamic mechanisms identified here provide a clear and reasonable explanation for their results.

Besides the presence of the sinuses, the current work indicates that the dimensional parameters of the system and the valve positioning have a strong influence on the valve performance. In the case of the sub-annular valve configuration, the positive start-up vortex forms high in the sinus, which therefore cannot provide an effective chamber to contain it. As a result, the vortex is pushed away by the main jet and escapes into the ascending root, confining the ejected flow onto one side of the root and producing a narrowing in the *vena contracta*. This reduces *d*_*vc*_ and *A*_*EO*_ by a 40% and 42%, respectively, from the physiological case, and produces a Δ*p* very similar to the sinusless configuration. However, the large space available at the valve outlet promotes the formation of a second vortex ring in the Valsalva sinuses, which supports closure of the valve leaflets, reducing *R*_*c*_ to the minimum value recorded in the reported cases (Table 1, Fig. 8). Globally, the energy loss is 77% higher than in the physiological configuration. Sub-annular configurations are very common in *in vitro* testing, where the prosthetic device is clamped in a housing component typically positioned below the mock aortic root. In fact, the mechanism of vortex migration and entrapment show similarities to those reported in the literature in several experimental studies (Lim et al., 1997, Lim et al., 1998, Lim et al., 2001). Our study indicates that the valve׳s axial positioning in the test rig can significantly affect the hemodynamics within the root, and therefore needs to be replicated with good care. From a clinical perspective, this set of experiments suggests that the current supra-annular implantation approach, recently preferred to infra-annular valve positioning, may result not just in a more favourable geometric orifice area, but also in significantly improved hemodynamics.

In the oversized root configuration, the STJ root diameter is increased by 16%. This increase impairs the ability of the sinus to confine the initial start-up vortex, which escapes downstream along the root, narrowing the jet. In particular, *d*_*vc*_ and *A*_*EO*_ are respectively 25% and 37% less than in the physiological configuration, and Δ*p* increases by 76%. After migration of the main vortex, a secondary negative vortex is formed within the sinus. This supports the valve closure, providing values of *R*_*c*_ similar to the optimal surgical configuration. As a result, *E*_*loss*_ is intermediate between those found for the optimal surgical and sinusless configurations. It is interesting and indicative to observe that, despite an aortic root cross sectional area 35% larger than in the other cases, this vortical mechanism results in a significant reduction in *A*_*EO*_ when compared to the optimal surgical configuration (Table 1), which is identical in valve size and position. Oversized root configurations are common in *in vitro* tests, where a universal root fits various valve sizes (Leo et al., 2005, Leo et al., 2006, Dasi et al., 2009, Saikrishnan et al., 2012); accordingly, the mechanism identified for this case is often observed in experimental studies. Also, similar operative modalities can be expected in clinical scenarios where the valve is under-sized, or the aortic root dilates due to pathological situations or, simply, ageing (Son et al., 2013, Vriz et al., 2014).

The interpretation of the current results has to take into account some approximations and experimental limitations.

Firstly the testing fluid solution is Newtonian, and has a lower dynamic viscosity than human blood. This may result in some departure of the flow from the physiological behaviour.

Also, the PIV system employed is 2D and phase-averaged, and therefore it cannot capture out-of-plane structures, nor reveal the cycle-to-cycle variation in flow patterns.

Finally, the mock roots were designed from an idealised physiological reference, without including the presence of the coronary ostia or the compliance of the vessels. Only one set of pressure and flow conditions was used throughout the experiments, reproducing typical healthy physiological conditions, at rest. Investigation of the effect of the root compliance, and further experiments for different operating conditions (*e.g*. different exercise conditions), could provide further insight into the valve flow mechanics, and will be the object of future studies.

However, the described limitations do not detract from the work, which clearly identifies different fluid mechanisms for the same valve, promoted by the specific system configuration.

## 5 Conclusions

The presented results clearly expose the fundamental role that the valve-root system plays on the functional mode and hydrodynamic performance of the left heart.

As expected, the configuration which replicated healthy native anatomies most closely produces hydrodynamic performances superior to all other cases. Any significant mismatch in the valve position, or variation in the geometric proportions of the root, determines major modifications of the fluid dynamics downstream of the valve, impairing the hydrodynamic performance. This can provide an explanation for the different vortical structures identified in the literature, and enlightens the experimental complications encountered when assessing the performance of prosthetic heart valves, even when this is only limited to the determination of the pressure drop, the effective orifice area, and regurgitant parameters.

The interaction of the valve device with the host anatomical roots should be taken into consideration when planning a surgical valve replacement, or when assessing valve performances *in vitro* and comparing data from different bench studies.

## Conflict of interest

None of the authors has any relationship with industry or financial associations that might pose a conflict of interest in connection with this work.

## Figures and Tables

**Table 1:** Hydrodynamic data for all configurations: mean value, ±SD and percentage difference with respect to physiological configuration.

					
		*i. Physiological configuration*	*ii. Optimal surgical config.*	*iii. Sinusless configuration*	*iv. Sub-annular configuration*	*v. Oversized root configuration*
***V***_***max***_ [m/s]⁎		**1.7**	**2.9** (+70%)	**3.0** (+75%)	**2.9** (+70%)	**2.8** (+65%)
***d***_***vc***_ [cm]*		**1.9**	**1.7** (−10%)	**1.4** (−25%)	**1.1** (−40%)	**1.4** (−25%)
***A***_***EO***_ [cm^2^]		**2.43**±0.02	**1.65**±0.01 (−32%)	**1.56**±0.02 (−36%)	**1.41**±0.01 (−42%)	**1.53**±0.01 (−37%)
**Δ*****p*** [mmHg]		**5.27**±0.08	**8.63**±0.13 (+64%)	**11.16**±0.23 (+110%)	**10.91**±0.07 (+101%)	**9.29**±0.15 (+76%)
***R***_***c***_ [ml]		**3.52**±0.16	**0.36**±0.08 (−90%)	**0.70**±0.09 (−80%)	**0.16**±0.04 (−95%)	**0.37**±0.11 (−89%)
***E***_***loss***_ [mJ]	*Forward*	**33.96**±1.1	**65.35**±1.01	**100.88**±2.32	**86.59**±1.56	**73.67**±1.71
	*Closing*	**15.18**±0.56	**1.28**±0.22	**2.74**±0.29	**0.50**±0.12	**1.57**±0.41
	***Total***	**49.14**±13.52	**66.63**±11.25 (+36%)	**103.62**±15.69 (+111%)	**87.09**±1.52 (+77%)	**75.24**±13.19 (+53%)

⁎ Values computed at instant B.
