# Supplementary material for: Physiological vortices in the sinuses of Valsalva: An in vitro approach for bio-prosthetic valves
Source: J Biomech. 2016 Sep 6;49(13):2635–43. doi: 10.1016/j.jbiomech.2016.05.027 (PMC5061069; doi:10.1016/j.jbiomech.2016.05.027)
Supplement: Supplementary file 1 — Supplementary material [file mmc1.docx]

**Appendix – Details on Testing Instrumentation**

Pressure catheters (Millar Mikro-Tip^®^, Millar Inc., USA) with resolution of ± 0.001 mmHg were used in the pulse duplicator to measure the pressure upstream and downstream of the aortic valve. Instantaneous volume flowrate was acquired at the valve inflow with an electromagnetic flowmeter (Carolina Medical Model FM501, Carolina Medical Electronics Inc., USA), with sensitivity of ± 0.1 ml/s and acquisition frequency of 256 samples per heart cycle. The resultant pressures and flowrate were used to estimate the effective orifice area and the closing volume, in compliance with the international standard (ISO 5840:2009) The energy losses were calculated in accordance with Leefe and Gentle (*J. Biomed. Eng.* 9 April 1987, pp. 121-7), over an average of 20 cycles for each experimental setup (first and last ten cycles of corresponding PIV data). A small amount of sodium thiophosphate was mixed into the water-KI solution to make it completely transparent. The resultant clear, colourless solution had the same dynamic viscosity of saline water at 37 °C (*η* = 1∙10-3 Pa×s).

For the PIV, neutrally buoyant hollow glass particles (Dantec Dynamics HGS-10, nominal diameter 10 µm) were used to seed the flow, and a cylindrical lens laser (dual cavities YAG laser, 70 mJ -x2- at 15 Hz, 532 nm, 1.5 mm maximum laser thickness) was used to illuminate the planar measurement region. A high speed camera (TSI PowerView™ Plus 4MP) captured the instantaneous positions of the seeding particles at the required instant, with a LaserPulse™ Synchronizer (Model 610036, TSI Inc., US, with 1 ns of time resolution) assuring accurate external triggering for phase-locked velocity measurements.

Depending on the instant within the cycle being considered (as detailed in the Materials and Methods section), the time interval between consecutive frames was varied in the range of *∆t* = 50-400 µs; this corresponds to a frequency of the dual cavity laser varying from 2.5 to 20.0 kHz, allowing full identification of slow and fast flow structures. The velocity vectors from the PIV images were calculated through Insight 4G^™^ (TSI Inc., US), using an adaptive correlation algorithm corresponding to a final spatial resolution of 400 µm. The acquired images were processed using a recursive Nyquist grid (with interrogation region evolving from 64×64 to 32×32 pixels and overlap of 50%), plus a fast Fourier transform correlation. Tecplot™ (Tecplot Inc., US) was used to compute and visualise the streamlines. The velocity fields were obtained by averaging 100 instantaneous flow fields (2 laser pulses at each instant for 100 cardiac cycles). This corresponds to a standard deviation below 2.6%.
